# Supplementary material for: Pressure-induced monotonic enhancement of Tc to over 30 K in superconducting Pr0.82Sr0.18NiO2 thin films
Source: Nat Commun. 2022 Jul 28;13:4367. doi: 10.1038/s41467-022-32065-x (PMC9334608; doi:10.1038/s41467-022-32065-x)
Supplement: Supplementary file 1 — Supplementary Information [file 41467_2022_32065_MOESM1_ESM.pdf]

Supplementary Information

Pressure-induced monotonic enhancement of T_c to over 30 K in the superconducting $Pr_{0.82}Sr_{0.18}NiO_2$ thin films

N. N. Wang[1,2=], M. W. Yang[1,2=], Z. Yang[1,2=], K. Y. Chen[1,2], H. Zhang[1,2], Q. H. Zhang[1,2], Z. H. Zhu[1,2], Y. Uwatoko[3], L. Gu[1,2], X. L. Dong[1,2], J. P. Sun[1,2*], K. J. Jin[1,2*] and J.-G. Cheng[1,2*]

[1]*Beijing National Laboratory for Condensed Matter Physics and Institute of Physics, Chinese Academy of Sciences, Beijing 100190, China*

[2]*School of Physical Sciences, University of Chinese Academy of Sciences, Beijing 100190, China*

[3]*Institute for Solid State Physics, University of Tokyo, Kashiwa, Chiba 277-8581, Japan*

= These authors contributed equally to this work.

Emails: jpsun@iphy.ac.cn; kjjin@iphy.ac.cn; jgcheng@iphy.ac.cn

As displayed below, we can see that the topotactic reduction time (Tr) and the amount of CaH_2 have strong influence on the superconducting transition of the infinite-layer $Pr_{0.82}Sr_{0.18}NiO_2$ thin films. In our study, we choose the best samples for high-pressure measurements, i.e. the samples obtained at T = 300°C, $m(CaH_2)$ = 0.21g, and Tr = 60 mins.

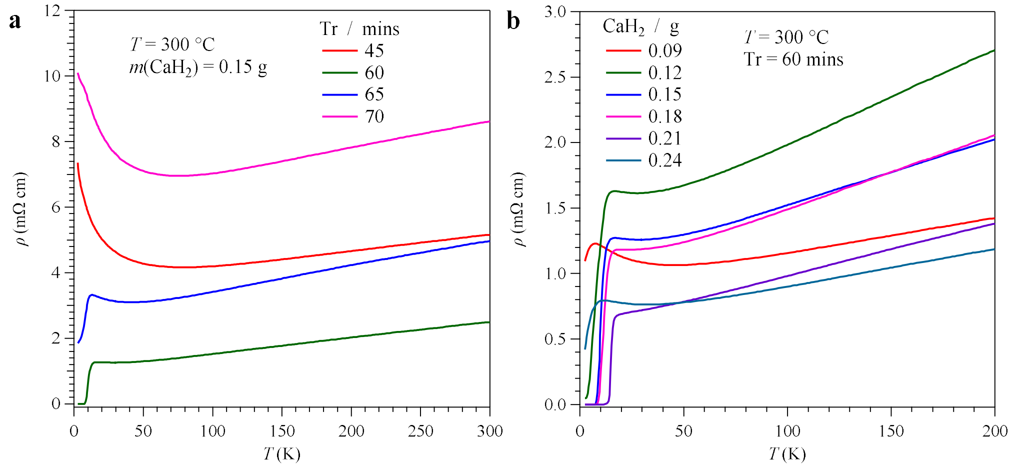

Supplementary Fig. 1 **Temperature dependence of resistivity for the $Pr_{0.82}Sr_{0.18}NiO_2$ thin films obtained at different conditions.** **a** T = 300°C, $m(CaH_2)$ = 0.15g, and Tr = 45-70 mins; **b** T = 300°C, $m(CaH_2)$ = 0.09-0.24 g, and Tr =60 mins.

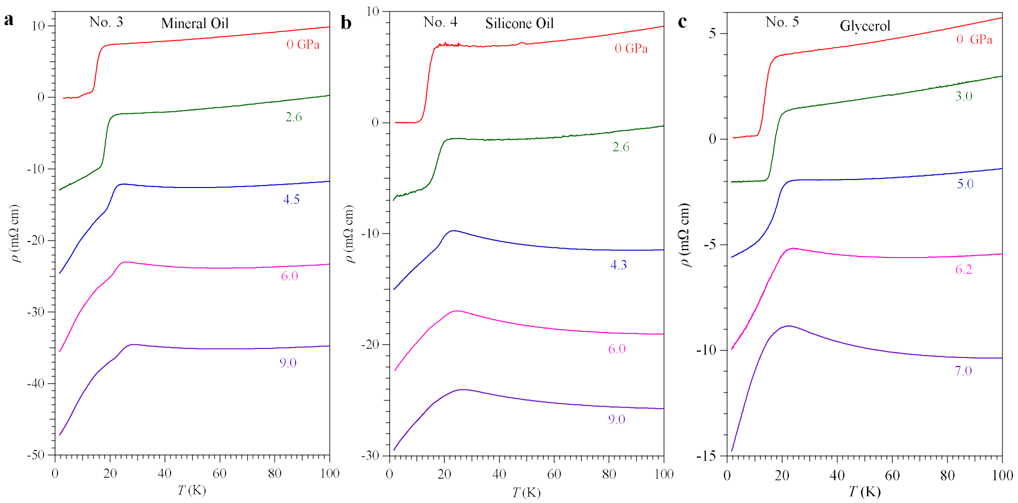

Supplementary Fig. 2 **High-pressure resistivity of $Pr_{0.82}Sr_{0.18}NiO_2$ thin films.** The resistivity $\rho(T)$ curves below 100 K **a** for No. 3 with mineral oil, **b** for No. 4 with silicone oil and **c** for No. 5 with glycerol, illustrating the variation of the superconducting transition temperatures with pressure. Except for data at 0 GPa, all other curves in **a**, **b** and **c** have been vertically shifted for clarity.

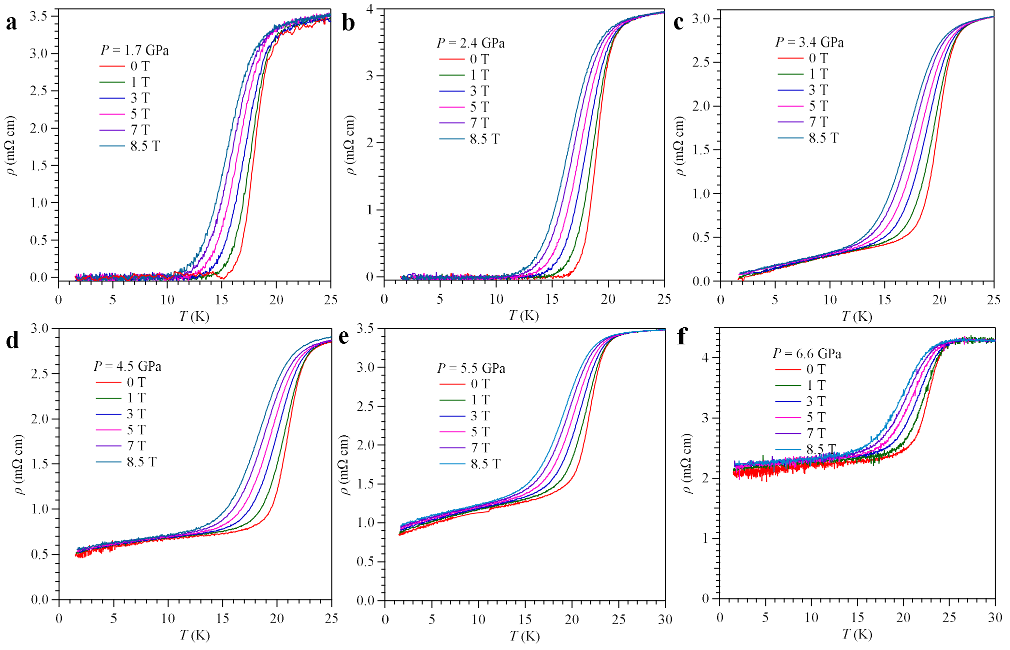

Supplementary Fig. 3 **Evolution of upper critical field of $Pr_{0.82}Sr_{0.18}NiO_2$ thin films.** Temperature dependences of the resistivity with magnetic fields up to 8.5 T at various pressures up to 6.6 GPa for sample No. 1.

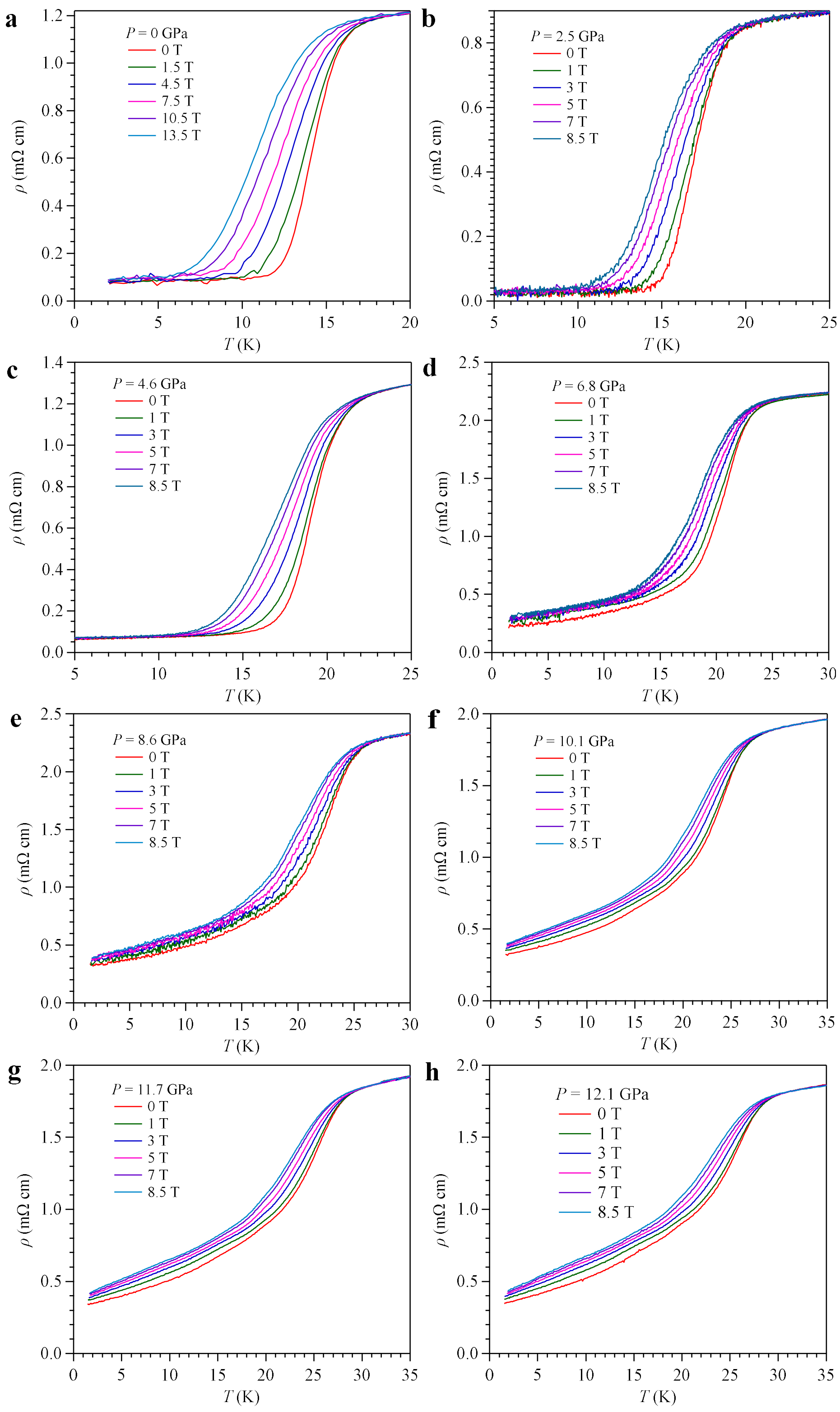

Supplementary Fig. 4 **Evolution of upper critical field of $Pr_{0.82}Sr_{0.18}NiO_2$ thin films.** Temperature dependences of the resistivity with magnetic fields up to 8.5 T at various pressures up to 12.1 GPa for sample No. 2.

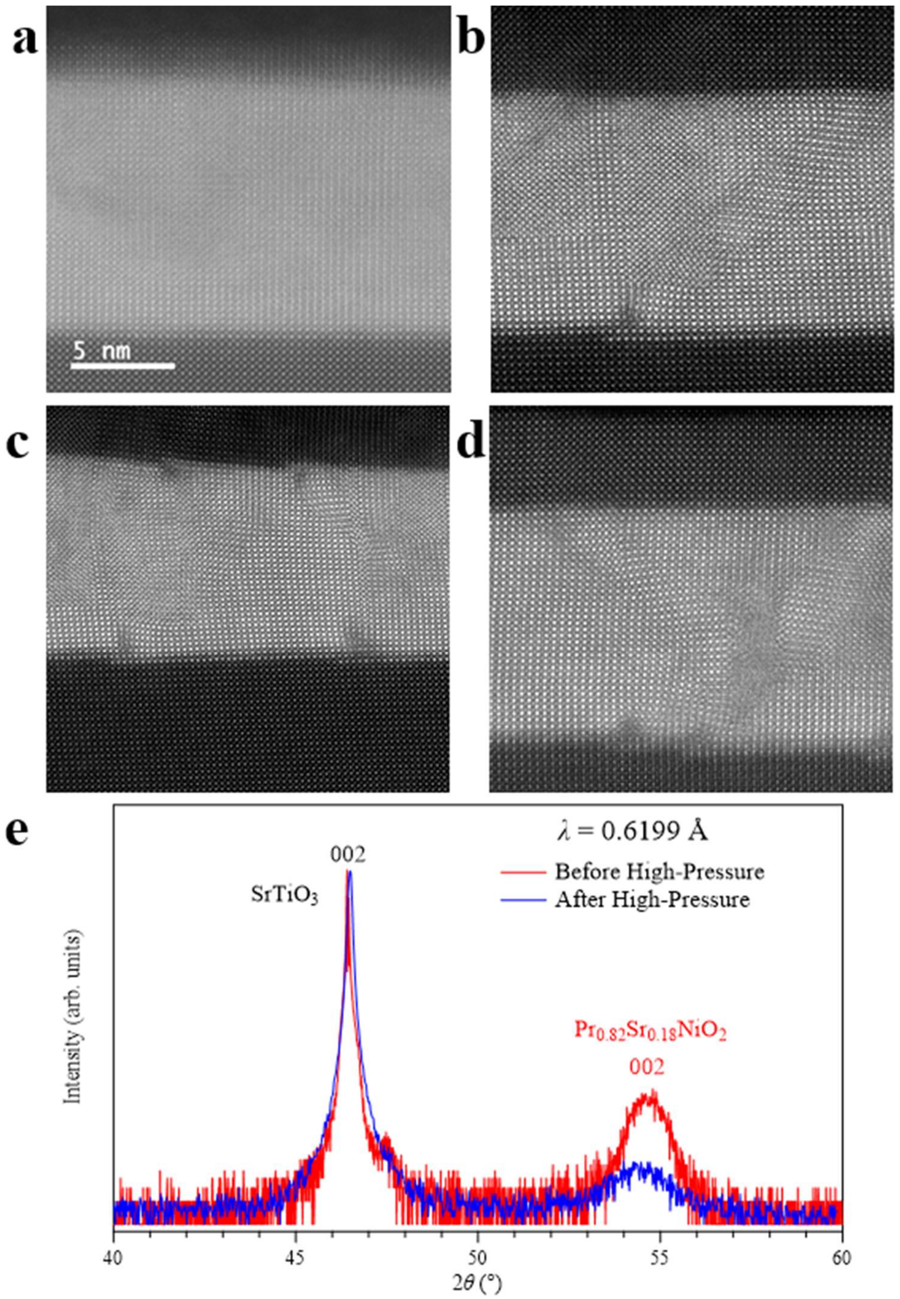

Supplementary Fig. 5 **Characterizations of $Pr_{0.82}Sr_{0.18}NiO_2$ thin films. a** The atomic-resolution STEM imaging of infinite-layer samples **a** before and **b-d** after the high-pressure resistivity measurements. **e** Synchrotron X-ray diffraction θ-2θ symmetric scans of infinite-layer $Pr_{0.82}Sr_{0.18}NiO_2$ thin film before and after high-pressure measurements with a wavelength of $\lambda = 0.6199$ Å.

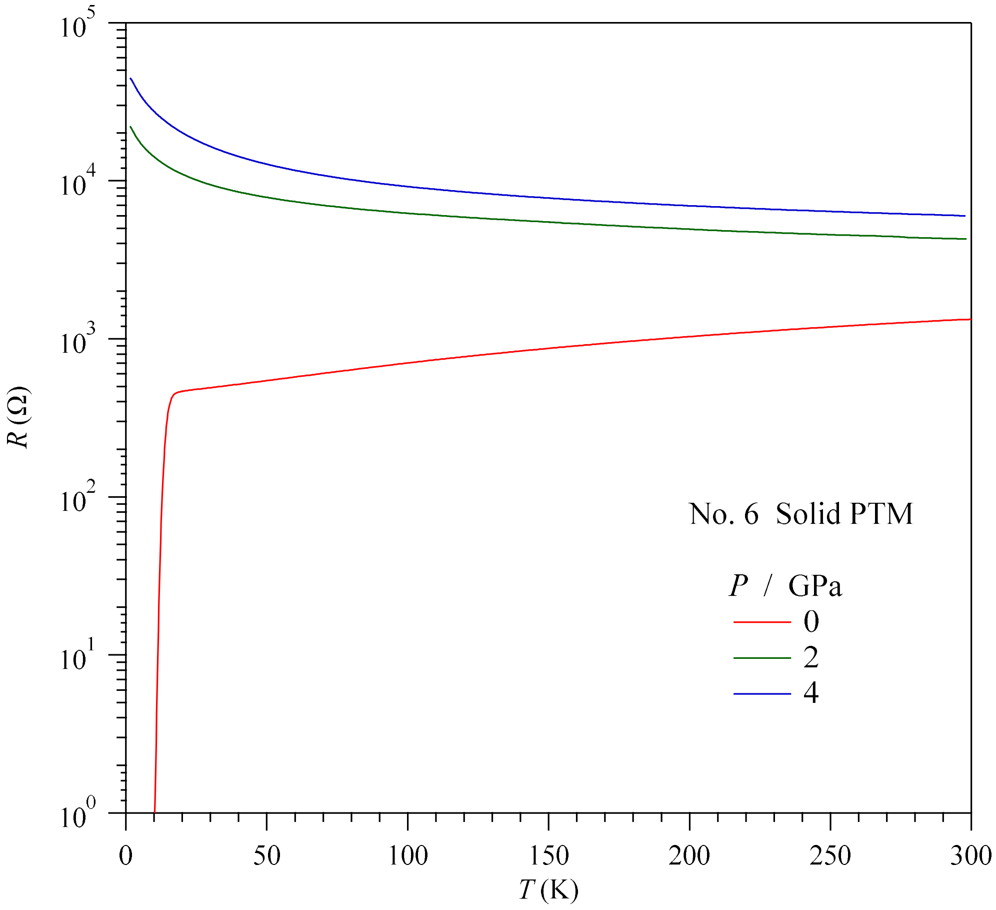

Supplementary Fig. 6 **High-pressure resistivity of $Pr_{0.82}Sr_{0.18}NiO_2$ thin films.** Temperature dependences of the resistance at 0, 2 and 4 GPa by employing the h-BN solid PTM.
